# Association between the use of e-cigarettes and heated tobacco products and asthma prevalence in adolescents: A secondary dataset analysis of the Korea Youth Risk Behavior Survey 2022–2024

**DOI:** 10.18332/tid/217962

**Published:** 2026-05-26

**Authors:** Jinyoung Kim, Sungkyu Lee

**Affiliations:** 1 Korea Center for Tobacco Control Research and Education, Seoul, Republic of Korea; 2 Graduate School of Public Health, Yonsei University, Seoul, Republic of Korea

**Keywords:** adolescents, e-cigarettes, heated tobacco products, asthma prevalence

## Abstract

**INTRODUCTION:**

Adolescent tobacco use is a major public health concern due to its adverse effects on respiratory health, including asthma. Novel tobacco products such as e-cigarettes (ECs) and heated tobacco products (HTPs) are increasingly used by youth, yet their respiratory health impacts remain insufficiently understood. This study examined associations between tobacco use patterns and asthma prevalence among Korean adolescents.

**METHODS:**

This secondary analysis used cross-sectional data from the nationally representative Korea Youth Risk Behavior Survey (KYRBS, 2022–2024), including 159383 students in grades 7–12. Current tobacco use (past 30 days) was classified into eight groups: non-users; exclusive users of combustible cigarettes (CCs), ECs, or HTPs; dual users; and triple users. Asthma was defined as a self-reported physician diagnosis within the past 12 months. Multivariable logistic regression adjusted for key covariates with survey weights applied.

**RESULTS:**

Asthma prevalence was 1.7%. Compared with non-users, adjusted odds ratios (AORs) were 1.34 (95% CI: 1.28–1.39) for CC-only users, 4.34 (95% CI: 4.17–4.51) for EC-only users, 9.16 (95% CI: 8.63–9.72) for HTP-only users, and 13.72 (95% CI: 13.25–14.22) for EC–HTP dual users. Triple users had an AOR of 5.15 (95% CI: 5.02–5.28). Secondhand smoke exposure, alcohol use, stress, and physical activity were also associated with asthma.

**CONCLUSIONS:**

Use of ECs and HTPs among Korean adolescents was associated with a higher prevalence of asthma. Concurrent EC and HTP use showed higher odds than single-product use, indicating a graded association across tobacco use patterns. These cross-sectional findings demonstrate robust associations but do not establish causality.

## INTRODUCTION

Asthma is among the most common chronic diseases in children and adolescents and is associated with substantial impacts on quality of life, school attendance, and healthcare utilization. Tobacco exposure during adolescence can impair lung development, promote airway inflammation, and increase vulnerability to respiratory diseases, including asthma and chronic obstructive pulmonary disease^1,2^.

Over the past decade, the tobacco product landscape has undergone profound changes with the introduction and rapid proliferation of novel products such as e-cigarettes (ECs) and heated tobacco products (HTPs). These alternatives to combustible cigarettes (CCs) are often promoted as reduced-risk products, although their health effects remain insufficiently characterized^3^. Studies have documented aggressive marketing campaigns targeting youth, including the use of fruity and mint flavors and sleek device designs, which lower perceived risks and facilitate initiation among never smokers^3^.

Despite marketing claims, growing toxicological and epidemiological evidence indicates that ECs and HTPs emit aerosols containing ultrafine particles, heavy metals, carbonyl compounds, and volatile organic compounds, which are implicated in airway epithelial injury, oxidative stress, and inflammation^4,5-7^. Furthermore, dual or poly-use of tobacco products, including combinations of CCs, ECs, and HTPs, is increasingly common among adolescents, potentially leading to additive or synergistic toxic exposures and heightened respiratory risks^8,9^.

In South Korea, national surveillance data reveal a rising trend in EC and HTP use among youth, contrasting with declining rates of conventional cigarette smoking^10^. However, regulatory measures remain insufficient to fully address the challenges posed by novel products, particularly with respect to flavored tobacco bans, sales restrictions, and advertising controls targeting adolescents. In contrast, countries such as Australia and Canada have implemented stringent policies to curb youth access to ECs and HTPs, including flavor prohibitions and comprehensive marketing restrictions^11,12^.

Given these shifting tobacco use patterns and emerging health concerns, robust epidemiological data examining the respiratory health impacts of EC and HTP use among adolescents are critically needed^13,14^. This study aims to fill this gap by analyzing nationally representative Korean data to assess associations between detailed tobacco use patterns and asthma prevalence, thereby informing evidence-based tobacco control policies.

## METHODS

### Study design and data source

This study is a secondary analysis of cross-sectional data from the Korea Youth Risk Behavior Web-based Survey (KYRBS), conducted annually from 2022 to 2024 by the Korea Disease Control and Prevention Agency (KDCA). The KYRBS employs a stratified, multistage, cluster sampling design to obtain a nationally representative sample of middle and high school students in South Korea. Data are collected via anonymous, self-administered online questionnaires, ensuring confidentiality and minimizing reporting bias. The overall student response rate for the KYRBS during 2022–2024 was approximately 92.2%^9^.

### Study population

The analytic sample included 159383 students in grades 7–12 who provided complete information on tobacco use, asthma diagnosis, and relevant covariates. Participants with missing or inconsistent responses for key variables were excluded from the analysis. Sampling weights were applied to account for the complex survey design and generate estimates representative of the national adolescent population.

### Measures

*Tobacco use patterns*

Current use of CCs, ECs, and HTPs was defined as any use within the past 30 days. Participants were categorized into eight mutually exclusive groups: non-users; CC-only; EC-only; HTP-only; CC+EC; CC+HTP; EC+HTP; and CC+EC+HTP^10^.

*Asthma prevalence*

Asthma was defined as a self-reported physician diagnosis within the past 12 months.

*Covariates*

Models adjusted for sex, grade, household income, academic performance, alcohol use, physical activity, breakfast habits, perceived stress, and secondhand smoke exposure at home. Breakfast habits were included as a proxy for overall health-related behaviors, which have been previously associated with respiratory and allergic outcomes in adolescents.

### Statistical analysis

All analyses accounted for the complex sampling design of the Korea Youth Risk Behavior Survey (KYRBS), including stratification, clustering, and survey weights, using the Complex Samples module in IBM SPSS Statistics for Windows, version 25.0^15^. Descriptive statistics were used to summarize demographic characteristics, tobacco use patterns, and asthma prevalence. Chi-squared tests were conducted to assess bivariate associations between tobacco use categories and asthma status.

Multivariable logistic regression models were fitted to adjusted associations between tobacco use patterns and asthma prevalence, with non-users serving as the reference group. Adjusted odds ratios (AORs) and 95% confidence intervals (CIs) are reported. All multivariable models were adjusted for sex, grade, household income, academic performance, alcohol use, physical activity, breakfast habits, perceived stress, and exposure to secondhand smoke at home. Interaction terms between tobacco product types were examined but excluded from the final models because they were not statistically significant. Model fit was assessed using the Hosmer–Lemeshow goodness-of-fit test, and multicollinearity was evaluated using variance inflation factors (VIF), with all values <2.5. Statistical significance was defined as p<0.05.

Although prevalence ratios can be estimated using alternative approaches such as robust Poisson regression, we applied multivariable logistic regression to ensure analytic consistency with prior KYRBS-based studies and comparability with existing literature. Given that odds ratios may overestimate prevalence ratios when the outcome is not rare, findings were interpreted primarily in terms of the direction and relative strength of associations rather than precise effect magnitude.

**Figure 1:**
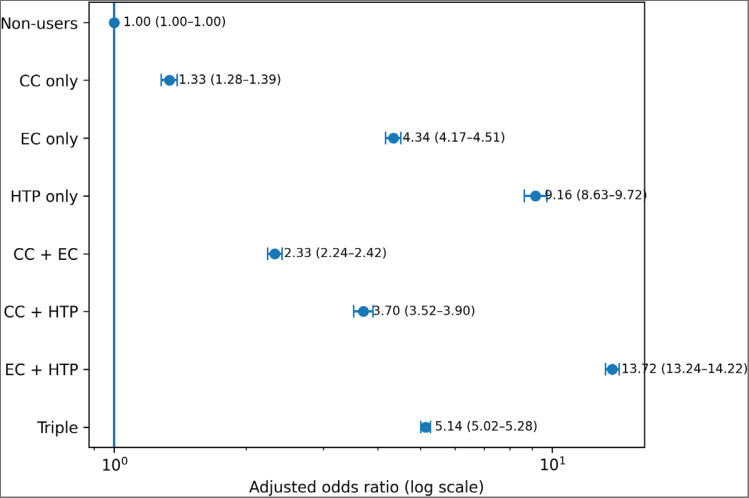
Adjusted odds ratio for asthma by tobacco use pattern

### Validity and reliability

The KYRBS employs standardized survey instruments that have been previously validated and shown to have acceptable reliability among Korean adolescents. In a national test–retest reliability study of the KYRBS questionnaire, core self-reported health behavior items, including tobacco use and physician-diagnosed asthma, demonstrated moderate to high reliability, with kappa ≥0.41 and several items with kappa ≥0.81^16^. The design and methodology of the KYRBS, including its complex sampling procedures and nationally representative framework, have been described in detail elsewhere^17^. Data collection follows rigorous protocols, including pilot testing, data quality monitoring, and standardized training for survey administrators, which support the external validity and generalizability of the findings.

This secondary analysis of publicly available, anonymized data was reviewed by the Institutional Review Board of the Ministry of Health and Welfare and was granted exemption from full ethical review (IRB No.: P01-202507-01-030).

## RESULTS

### Demographic and behavioral characteristics

Table 1 summarizes the demographic characteristics, tobacco use patterns, alcohol consumption, and asthma prevalence of the study population. Among the 159383 adolescents included in the analysis, the overall prevalence of physician-diagnosed asthma within the past 12 months was 1.7%.

**Table 1:** Demographic characteristics, tobacco use patterns, alcohol consumption, and asthma prevalence among Korean adolescents, KYRBS 2022–2024 (N=159383)

*Variables*	*Categories*	*n*	*Weighted %*
**Sex**	Boys	81256	51.5
	Girls	78127	48.5
**Grade**	7	28773	17.0
	8	28415	16.9
	9	28315	17.1
	10	26511	17.1
	11	24845	16.2
	12	22524	15.7
**Academic performance**	High (top)	60930	39.3
	Middle	46868	28.6
	Low (bottom)	51575	32.1
**Household economic status**	High	67441	45.3
	Middle	73555	43.9
	Low	18374	10.8
**Tobacco use behavior^†^**	Non-users	151531	95.0
	CC only	2200	1.4
	EC only	821	0.5
	HTP only	190	0.1
	CC + EC	1594	1.0
	CC + HTP	539	0.4
	EC + HTP	430	0.3
	CC + EC + HTP	2078	1.3
**Alcohol use**	Non-drinker	141675	86.7
	Current drinker	17708	13.3
**Asthma** (past 12 months)	No	156668	98.3
	Yes	2715	1.7

Values are presented as sample size (n) and weighted percentage (%), accounting for the complex survey design. The study population included adolescents in grades 7–12 enrolled in middle and high schools in South Korea. Asthma was defined as self-reported physician diagnosis within the past 12 months. Tobacco use behavior was classified based on any use within the past 30 days. CC: combustible cigarettes. EC: electronic cigarette. HTP: heated tobacco product. KYRBS: Korea Youth Risk Behavior Web-based Survey.

† Tobacco use behavior categories are mutually exclusive.

The sample comprised 51.5% boys and 48.5% girls, in grades 7–12. Academic performance was reported as high by 39.3% of participants, middle by 28.6%, and low by 32.1%. Household economic status was categorized as high in 45.3% of adolescents, middle in 43.9%, and low in 10.8%. With respect to tobacco use behavior, 95.0% of participants reported no use of tobacco products. Exclusive use of combustible cigarettes (CCs), electronic cigarettes (ECs), and heated tobacco products (HTPs) was reported by 1.4%, 0.5%, and 0.1% of adolescents, respectively. Dual use of ECs and HTPs accounted for 1.0% of the sample, and 1.3% reported concurrent use of all three tobacco products. In addition, 13.3% of students reported current alcohol consumption.

### Association between tobacco use and asthma

As shown in Table 2, significant differences in asthma prevalence were observed according to sex, grade, household economic status, perceived stress, breakfast skipping, physical activity, tobacco use behavior, exposure to secondhand smoke at home, and alcohol consumption (all p<0.001). Asthma prevalence was higher among boys than girls and was more common among adolescents from low-income households compared with those from high-income households. Adolescents reporting perceived stress, skipping breakfast, exposure to secondhand smoke at home, or current alcohol consumption showed a higher prevalence of asthma. Marked differences in asthma prevalence were also observed across tobacco use categories, with higher proportions of asthma among adolescents reporting use of ECs and HTPs, particularly among those using multiple tobacco products.

**Table 2:** Bivariate associations between selected factors and asthma prevalence among Korean adolescents, KYRBS 2022–2024 (N=159383)

*Variables*	*No Asthma* *(N=156668)*		*Asthma* *(N=2715)*		*Total* *(N=159383)*		*p*
	*n*	*wt%*	*n*	*wt%*	*n*	*wt%*	
**Sex**							
Boys	79711	51.4	1545	57.1	81256	51.5	<0.001
Girls	76957	48.6	1170	42.9	78127	48.5	
**Grade**							
7	28314	17.0	459	16.3	28773	17.0	<0.001
8	27968	16.9	447	15.3	27415	16.9	
9	27821	17.1	494	17.7	28315	17.1	
10	26089	17.1	422	15.9	26511	17.1	
11	24396	16.2	449	17.9	24845	16.2	
12	22080	15.7	444	16.9	22524	15.7	
**Household economic status**							
High	66280	43.3	1161	44.1	67441	43.3	<0.001
Middle	72428	45.7	1127	40.8	73555	45.7	
Low	17947	11.0	427	15.1	18374	11.0	
**Presence of stress**							
No	26300	16.6	468	17.7	26768	16.6	<0.001
Yes	130368	83.4	2247	82.3	132615	83.4	
**Skipping breakfast**							
No	51797	33.5	826	30.5	52650	33.4	<0.001
Yes	104868	66.5	1889	69.5	106757	66.6	
**Physical activity**							
Yes	27248	16.9	563	19.9	27811	16.9	<0.001
No	129420	83.1	2152	80.1	131572	83.1	
**Tobacco use behavior^†^**							
Non-users	149315	95.2	2216	81.2	151531	95.0	<0.001
CC only	2143	1.4	57	1.8	2200	1.4	
EC only	774	0.5	47	2.1	821	0.5	
HTP only	162	0.1	28	1.0	190	0.1	
CC + EC	1530	1.0	64	2.3	1594	1.0	
CC + HTP	507	0.3	32	1.3	539	0.4	
EC + HTP	342	0.2	88	3.3	430	0.3	
CC + EC + HTP	1895	1.2	183	6.9	2078	1.3	
**Exposure to secondhand smoke at home**							
Yes	123332	20.5	1853	31.3	125185	20.7	<0.001
No	123332	79.5	1853	68.7	34198	79.3	
Alcohol use							
Non-drinker	139517	88.9	2158	78.9	141675	88.7	<0.001
Current drinker	17151	11.1	557	21.1	17708	11.3	

Values are presented as sample size (n) and weighted percentage (%). P-values were calculated using the chi-squared test to account for the complex sampling design of the Korea Youth Risk Behavior Survey. Asthma was defined as self-reported physician diagnosis within the past 12 months. Tobacco use behavior was classified based on any use within the past 30 days. CC: combustible cigarettes. EC: electronic cigarette. HTP: heated tobacco product.

† Tobacco use behavior categories are mutually exclusive.

### Multivariable logistic regression analysis

Results of the multivariable logistic regression analyses are presented in Table 3. After adjustment for sex, grade, household economic status, perceived stress, breakfast skipping, physical activity, tobacco use behavior, exposure to secondhand smoke at home, and alcohol use, tobacco use patterns were strongly associated with asthma prevalence.

**Table 3:** Multivariable logistic regression analysis of factors associated with asthma prevalence among Korean adolescents, KYRBS 2022–2024 (N=159383)

*Variables*	*AOR*	*95% CI*
**Sex**		
Boys (ref.)	1.00	
Girls	1.16***	1.14–1.17
**Grade**		
7 (ref.)	1.00	
8	1.10***	1.08–1.13
9	0.99	0.97–1.01
10	1.09***	1.07–1.11
11	0.96***	0.95–0.98
12	1.06***	1.04–1.08
**Household economic status**		
High (ref.)	1.00	
Middle	0.89***	0.88–0.90
Low	1.14***	1.12–1.16
**Presence of stress**		
Yes (ref.)	1.00	
No	1.06***	1.04–1.07
**Skipping breakfast**		
No (ref.)	1.00	
Yes	1.04***	1.03–1.06
**Physical activity**		
No (ref.)	1.00	
Yes	1.16***	1.14–1.18
**Tobacco use behavior^†^**		
Non-users (ref.)	1.00	
CC only	1.34	1.28–1.39
EC only	4.34***	4.17–4.51
HTP only	9.16***	8.63–9.72
CC + EC	2.33***	2.24–2.42
CC + HTP	3.70***	3.52–3.90
EC + HTP	13.72***	13.25–14.22
CC + EC + HTP	5.15***	5.02–5.28
**Exposure to secondhand smoke at home**		
No (ref.)	1.00	
Yes	1.49***	1.47–1.51
**Alcohol use**		
Non-drinker (ref.)	1.00	
Current drinker	1.16***	1.14–1.18

Values are presented as adjusted odds ratios (AORs) with 95% confidence intervals (CIs). All models were adjusted for sex, grade level, household economic status, perceived stress, breakfast skipping, physical activity, tobacco use behavior, exposure to secondhand smoke at home, and alcohol use. KYRBS: Korea Youth Risk Behavior Web-based Survey. CC: combustible cigarettes. EC: electronic cigarette. HTP: heated tobacco product.

† Tobacco use behavior was classified into mutually exclusive categories based on use within the past 30 days.

*** p<0.001.

Compared with non-users, the adjusted odds ratios (AORs) for asthma were 1.34 (95% CI: 1.28–1.39) for CC-only users, 4.34 (95% CI: 4.17–4.51) for EC-only users, and 9.16 (95% CI: 8.63–9.72) for HTP-only users. Dual use of ECs and HTPs was associated with the highest odds of asthma (AOR=13.72; 95% CI: 13.25–14.22), while triple use of CCs, ECs, and HTPs was also associated with elevated odds (AOR=5.15; 95 % CI : 5.02–5.28).

In addition to tobacco use behavior, exposure to secondhand smoke at home (AOR=1.49; 95% CI: 1.47–1.51), current alcohol consumption (AOR=1.16; 95% CI: 1.14–1.18), perceived stress (AOR=1.06; 95% CI: 1.04–1.07), and physical activity (AOR=1.16; 95% CI: 1.14–1.18) were associated with higher odds of asthma.

These results indicate a graded association between patterns of tobacco product use and asthma prevalence among Korean adolescents. In particular, concurrent use of ECs and HTPs was associated with higher odds of asthma compared with single-product use. This pattern suggests a potential cumulative burden associated with exposure to multiple aerosol-generating products, rather than a statistically defined interaction effect. These findings underscore the substantial respiratory health burden associated with emerging tobacco products and secondhand smoke exposure among youth.

## DISCUSSION

This study is a novel, large-scale investigation that examines the association between EC and HTP use and asthma prevalence among adolescents, using nationally representative data. The findings showed that adolescents who exclusively used HTPs and those who were dual users of ECs and HTPs had markedly higher odds of asthma compared with non-users. In particular, concurrent use of ECs and HTPs was associated with a substantially greater likelihood of reporting asthma than single-product use, suggesting that combined aerosol exposure from both products may enhance airway inflammatory responses and trigger immunologic hypersensitivity reactions^2,10,13^.

According to previous studies, EC aerosols contain fine particulate matter (PM2.5), nicotine, propylene glycol, glycerin, flavoring chemicals, and heavy metals such as lead, chromium, and nickel. These substances have been reported to cause airway epithelial injury, oxidative stress, and increased secretion of inflammatory cytokines, including IL-6, IL-8, and TNF-α^3,4,14^. Because adolescents’ respiratory systems are structurally and immunologically immature, they are more susceptible to aerosol-induced inflammation than adults^4,14^.

In the case of HTPs, despite marketing claims that these products are ‘heated, not burned’, aerosols generated at temperatures exceeding 350°C have been found to contain carcinogenic and irritant compounds such as formaldehyde, acetaldehyde, and acrolein^5-7^. These chemicals can cause oxidative damage to the bronchial mucosa and promote eosinophilic inflammation, thereby exacerbating the pathophysiological processes of asthma. Furthermore, studies have reported that the levels of carbon monoxide and nicotine metabolites after HTP use are comparable to – or in some cases even higher than – those observed among conventional cigarette smokers, contradicting tobacco industry claims that these products are ‘less harmful’ alternatives^6^.

The elevated asthma risk observed among dual EC–HTP users in this study may be explained by potential interactions between toxicants and exposure pathways of the two products. For example, inhaling HTP aerosols after EC use could expose an already irritated airway to additional harmful compounds, amplifying inflammatory responses^2,5,14^. Repeated exposure may increase airway hyperreactivity and establish a chronic inflammatory milieu that exacerbates asthma symptoms over time.

Although the KYRBS employs nationally validated survey instruments with demonstrated reliability, reliance on self-reported measures for EC and HTP use and physician-diagnosed asthma may still be subject to reporting and recall bias, potentially leading to non-differential misclassification. In addition, the KYRBS does not collect information on the daily quantity of tobacco use, duration of use, nicotine dependence, or former CC use, which may result in residual confounding and limit a more detailed assessment of exposure–response relationships.

In addition, this study confirmed that secondhand smoke exposure, alcohol consumption, and stress were associated with increased asthma risk, whereas regular physical activity served as a protective factor^10^. These findings highlight the importance of addressing not only tobacco use but also daily health behaviors and psychosocial stressors in promoting respiratory health among adolescents.

In summary, the use of ECs and HTPs among adolescents poses significant respiratory and immunologic risks beyond nicotine dependence^2,4,14,15^. Simultaneous use of multiple aerosol-generating tobacco products may be associated with a greater cumulative exposure burden, potentially increasing susceptibility to respiratory harm. Although causal inference cannot be established given the cross-sectional design, these findings highlight the importance of considering youth-focused tobacco control efforts that address emerging tobacco products, including measures related to access, marketing, and prevention education.

### Limitations

Several limitations of this study should be acknowledged. First, the cross-sectional design precludes causal inference regarding the observed associations between tobacco product use and asthma. Second, both asthma status and tobacco use were assessed using self-reported measures, which may be subject to misclassification bias due to recall error or social desirability, potentially leading to non-differential misclassification. Third, although we adjusted for a wide range of sociodemographic, behavioral, and environmental factors, residual confounding by unmeasured variables – such as intensity and duration of tobacco use, nicotine dependence, or prior combustible cigarette use – cannot be excluded. In addition, the findings may not be fully generalizable to adolescents who are not enrolled in school or to settings with different cultural and regulatory contexts. Finally, reverse causality cannot be ruled out with this cross-sectional design. Despite these limitations, the present study provides large-scale, nationally representative evidence of an association between emerging tobacco product use and asthma prevalence among adolescents, underscoring the importance of continued surveillance and youth-focused tobacco control and prevention efforts.

## CONCLUSIONS

Using nationally representative data from the Korea Youth Risk Behavior Web-based Survey (KYRBS), this study found that EC and HTP use among Korean adolescents was significantly associated with a higher likelihood of reporting asthma. In particular, adolescents who exclusively used HTPs and those who concurrently used ECs and HTPs exhibited higher odds of asthma compared with non-users or single-product users, indicating a graded association across patterns of tobacco product use. These findings extend existing evidence on the respiratory health risks of emerging tobacco products by highlighting the potential public health implications of multiple product use during adolescence, a critical period of lung development. Although causal relationships cannot be inferred due to the cross-sectional design, the observed associations underscore the importance of continued surveillance of novel tobacco product use among youth and the need to strengthen youth-focused tobacco control and prevention strategies that address both individual products and patterns of combined use.

## Supplementary Material



## Data Availability

The data supporting this research are available from the authors on reasonable request.
